# Diagnosis and treatment of occupational burnout in the Swiss outpatient sector: A national survey of healthcare professionals’ attributes and attitudes

**DOI:** 10.1371/journal.pone.0294834

**Published:** 2024-12-11

**Authors:** Irina Guseva Canu, Roger Getzmann, Yara Shoman, Fulvia Rota, Stéphane Saillant, Roland von Känel, Christine Cohidon, Catherine Lazor-Blanchet, Lysiane Rochat, Rafaël Weissbrodt, Nadia Droz, Anny Wahlen

**Affiliations:** 1 Department of Environmental and Occupational Health, Center for Primary Care and Public Health (Unisanté), University of Lausanne, Lausanne, Switzerland; 2 Swiss Society for Psychiatry and Psychotherapy, Bern, Switzerland; 3 Centre Neuchâtelois de Psychiatrie (CNP), Neuchatel, Switzerland; 4 University of Lausanne, Lausanne, Switzerland; 5 Department of Consultation-Liaison Psychiatry and Psychosomatic Medicine, University Hospital Zurich, University of Zurich, Zürich, Switzerland; 6 Department of Family Medicine, Center for Primary Care and Public Health (Unisanté), University of Lausanne, Lausanne, Switzerland; 7 Occupational Health Service, Lausanne University Hospital, Lausanne, Switzerland; 8 School of health sciences, HES-SO Valais-Wallis, Sion, Switzerland; 9 PSY4WORK.ch, the Swiss Association of work & organization psychologists, Lausanne, Switzerland; St John’s University, UNITED STATES OF AMERICA

## Abstract

We aimed to describe the attributes and attitudes of Swiss health professionals who treat persons with occupational burnout (POB) in the outpatient sector and explore associated determinants. The study design was descriptive cross-sectional survey, distributed to the 16,883 general practitioners (GP), psychiatrist-psychotherapists (PP), occupational physicians (OP) and psychologists registered in the Swiss Medical Association, the Swiss Federation of Psychologists, and other specialized associations. Using an online questionnaire, we identified professionals who consult and treat POB, their attributes, volume of POB consultations, diagnostics and treatment modalities and outcomes (OB severity, average proportion of POB who returned to work and who relapsed). Multinomial regression analysis was conducted to identify attributes associated with these outcomes. Among 3216 respondents, 2951 reported to consult POB, and 1130 (713 physicians and 410 psychologists) to treat them. POB consultations constitute 5 to 25% of professionals’ consultations, which varies across professionals’ specialties and specializations and geographic regions. The profile of POB consulted also differs across professionals. Work psychologists reported more often consulting POB at early OB stage, GPs mostly reported having patients with moderate OB, while PPs reported having the largest proportion of patients with severe OB. The treatment practices depend on OB severity but neither latter nor former was associated with the proportion of relapsed POB or POB who return to work. Physicians with waiting time >3 months reported more often having a higher proportion of relapsed patients. Since the study had an exploratory nature using a cross-sectional survey design and aggregated outcomes, these findings should be considered as first descriptive data, motivating further research.

## Introduction

### Background

Since May 2019, burnout is recognized as an occupational phenomenon resulting from “chronic stress at the workplace that has not been successfully managed” [1]. In the 11th revision of the International Classification of Diseases (ICD-11), burnout is classified among “Factors influencing health status or contact with health services”, i.e., reasons for using health services, which are not ascribed as a disease [2]. In its advanced stage, burnout shares several symptoms with depression, but burnout is mostly seen as a risk factor for depression or a mediator in the relationship between exposure to job stress and depression [3–5]. Conversely to depression, established as a leading cause of disability worldwide and a major contributor to the overall global burden of disease [6, 7], burnout is recognized as a disease only in few countries [8]. Despite a recently launched harmonization effort at the European level, with an introduction of a more specific, consensus-based term “occupational burnout” (OB) and its harmonized definition [8, 9], the generic term “burnout” is still widely used in the general media and literature. For the sake of consistency, we will use the terms employed in the original studies (e.g., burnout, emotional exhaustion) when referring to the published literature, and the term OB when reporting methods and findings of the current study. The latter was defined as follows: “in workers, OB is the state of physical and psychological exhaustion due to prolonged exposure to work-related problems” [9].

There are still no standardized and internationally accepted criteria for detection and diagnosis of OB, although several self-assessment and one hetero-assessment instruments have been developed, with more or less established psychometric validity [10–12]. In countries, where burnout is diagnosed using a specific code (e.g., Z73 in the ICD-10 or Q85 in the ICD-11), the reported prevalence ranges between 3 and 6% [13]. There is little scientific information on the treatment of burnout [14, 15], which depends on its understanding, whether it is regarded as independent disease, as preliminary stage of a depression or as a comorbidity of depression [16–18]. Therapies which are used for the treatment of burnout includes psychotherapy, especially cognitive behavioral therapy (CBT), phytotherapy, physiotherapy, adjuvant pharmacotherapy and complementary treatments like music therapy or body-mind therapies [18–20]. However little is known on their implementation and effectiveness, even in countries with established guidelines on burnout detection and treatment, like the Netherlands [21, 22], France [23], Belgium [24], and Switzerland [4, 5].

Despite the existing guidelines, important variations in diagnostic and medical practices have been observed among general practitioners and occupational physicians in a cantonal outpatient center in Switzerland [25]. Although OB is not considered a disease in Switzerland, its prevalence was estimated at 4% based on the meta-analysis of the available reports published before the COVID-19 pandemic [13]. Since the COVID-19 pandemic, burnout was reported at unprecedented rates in virtually all countries [26–28]. In Switzerland, the 2022 Job Stress Index indicated that the proportion of Swiss workers at risk of burnout exceeded 30% [29]. However, a longitudinal study showed that conditions at work, as well as most health-related variables, including emotional exhaustion, did not deteriorate [30]. While debates regarding its status and recognition continue, a non-negligeable part of the active population with severe, clinical burnout needs medical treatment. In a prior qualitative study, we identified that health professionals who are the most concerned with POB in their clinical practice are general physicians (GPs), occupational physicians (OPs), psychiatrists-psychotherapists (PPs), psychologists, and occupational nurses [31. While for physicians the persons with OB (POB) are patients, in psychology practice POB are considered as clients.

In this study, the POB means the person that the targeted health professional has characterized as having OB based on his/her professional judgment. There are no formal criteria for diagnosing burnout in Switzerland. Since the ICD-11 has not yet been implemented in the Swiss healthcare system, physicians continue to use the ICD-10 code Z73.0, which refers to "Burn-out." The diagnosis is typically made based on a thorough history, particularly in the context of chronic workplace stress leading to emotional exhaustion, reduced work efficacy, and depersonalization from one’s work. Comorbid psychiatric diagnoses, such as depression, anxiety disorders, and adjustment disorders, are assessed and coded, as these are required for financial compensation by insurance companies if the patient is referred to hospital treatment for burnout. The diagnoses usually officially reported for OB situations are adjustment disorder with or without precision of OB and mild or moderate depression with or without precision of OB. The diagnosis of mixed anxiety and depressive disorder with or without precision of OB is reported more rarely. When these diagnoses must be given to a health insurer, they have an impact on the length of sick leave duration tolerated by the insurer, the first two being shorter.

### Study purpose

Considering this national and international context, our main objective was to explore the characteristics and activities of Swiss health professionals who treat POB in the Swiss outpatient sector. The study was set up to describe their attributes and attitudes with respect to the diagnosis and treatment of POB and explore their potential determinants.

### Methods

#### Study design

We applied the design of a descriptive cross-sectional survey [32] and followed the Consensus-Based Checklist for Reporting of Survey Studies (CROSS) [33].

#### Data collection

To collect the necessary data, we construed an electronic questionnaire based on the questionnaire developed by Droz and Wahlen [34] and the information obtained from our qualitative study described in previous reports [31, 35]. In order to reduce the number and complexity of the questions, the questionnaire was adaptive, with certain questions conditionally displayed based on the responses to other questions. Most questions were multiple choice questions (MCQ) and included a non-response option “Impossible to specify” or “I don’t know”. The questionnaire was thematically divided into four parts: 1-demographic and practice-related data of the participants (6 MCQ, and 2 open questions); 2-data on the burnout definition and detection (10 MCQ and 8 open questions), where we asked the respondents to provide their working definition of the early, moderate and advanced/severe stages of OB, the characteristic symptoms of each stage and differential diagnoses they do to discriminate OB; 3-data on the treatment of POBs (12 MCQ and 3 open questions including the proportion of treated POB who had been able to return to work, and the proportion of treated POB who relapsed); and 4-information on needs and suggestions for practice improvement (5 MCQ and 2 open questions). Two versions of the questionnaire were developed separately for physicians and psychologists. The psychologists’ version included three additional questions on factors related to individual dispositions, personal and social context, and working conditions and professional activity. These questions were not addressed to physicians, as the survey took place at the height of the 1^st^ anti-COVID vaccination campaign, this additional constraint on doctors had to be considered. A French version of the questionnaire was first developed. This version was examined, tested, and validated by the project’s scientific committee including 6 psychologists, 6 physicians representing the specialty of the target group, 1 pharmacologist, 2 medical sociologists, and 1 epidemiologist. It was also presented to the advisory committee. Italian and German versions of the questionnaire were then developed and tested by members of the project’s scientific committee and by Italian- and German-speaking Unisanté staff. The full questionnaire as well as more details on its creation and testing were published previously [35].

The electronic version of the questionnaire was implemented using REDCap, an electronic data capture tool hosted at Unisanté [36, 37]. REDCap is a secure, web-based software platform providing an intuitive interface for validated data capture, audit trails for tracking data manipulation and automated export procedures for seamless data downloads to common statistical packages. An e-mail and a postal letter containing the study information and the QR code for participation were sent to eligible professionals either by the professional associations (described below) or by the research team. The participation was voluntary and anonymous. Data entry fields had validation mechanisms and it was made sure that participants only participated once. The questionnaire was promoted through various means, including professional associations, press releases and a web page dedicated to the study. One reminder was sent using the same distribution channels.

The collected data were automatically transferred to a secure and access-restricted server. The data did not contain any personal identifiers.

#### Target population and study sample

The target population of the study was Swiss health professionals who can treat POB in the outpatient setting, i.e. physicians, psychologists and occupational nurses (Total n = 16’883). The lists and contact information for these professionals were obtained from the Swiss Medical Association (FMH), the Swiss Federation of Psychologists (FSP), the Swiss Society for Psychiatry and Psychotherapy (SSPP), the Association of Organization and Work Psychologists (PSY4WORK.CH), and the Swiss Association of Occupational Health Nurses (ASIST). Participants who were not confronted with POB were asked to leave the questionnaire after their demographic data had been collected. Participants who did not treat POB themselves were asked to leave the questionnaire before questions about treatment options were asked.

The study sample consisted of 3’216 health care professionals who participated between April 7 and July 20, 2021 in the electronic survey. Since the participation was completely anonymous, the need for consent was waived by the ethics committee (CER-VD BASEC-Nr. Req-2021-01156). Table 1 provides the number of respondents and response rates per profession and medical specialty.

**Table 1:** Reponse rate per profession and medical specialty in the STBOS-VD survey.

Profession	Target population	Number of respondents	Response rate (%)
Physiciens	10 272	1 723	17
General practitioners	7 227	874	12
Psychiatrists	2 968	657	22
Psychologists	6 514	1 326	20
Occupational health nurses	97	39	40
Other	-	128	-
Total	16 883	3 216	19

#### Data processing and statistical analysis

Data were accessed for research purposes from the November 1^st^, 2021 through the April 30^th^ 2023. First, we checked and recoded variables to reduce the number of categories. For instance, a new 7-class variable “Region of practice” was created: 1-“Lake Geneva region (VD, VS, GE)” 2-“Espace Mittelland (BE, FR, SO, NE, JU)” 3-“Northwestern Switzerland (BS, BL, AG)” 4-“Zurich (ZH)” 5- “Eastern Switzerland (GL, SH, AR, AI, SG, GR, TG)” 6. “Central Switzerland (LU, UR, SZ, OW, NW, ZG)” 7-“Ticino (TI)”. Furthermore, 18 dichotomic variables (yes/no) for options of OB treatment were transformed in a 7-class variables “treatment options chosen”, for physicians and for psychologists, respectively. Finally, we dichotomized the outcome variables “Prognostic beliefs” as “Yes, OB can absolutely be healed” versus “OB can sometimes or never completely be healed”; “Severity of burnout” as “Mostly mild or moderate burnout” versus “Mostly severe burnout”; “Proportion of patients with relapse” as “< = 25% patients who relapsed” versus “>25% patients who relapsed”; and “Proportion of patients fit for work” as “< = 75% patients that are fit for work” versus “>75% patients that are fit for work”.

The statistical analysis was conducted according to a pre-defined statistical analysis plan (SAP), where we defined each research question along with the statistical model and variables to answer it. This SAP was developed following a thorough descriptive analysis of the collected data and discussion with the project’s scientific committee [35]. The SAP is available at the Unisante data depository (https://doi.org/10.16909/dataset/42).

To reduce the risk of type I error, also known as a false positive, we limited our analysis to ten questions as follows: Q1-Who among Swiss health professionals are confronted with POB in the outpatient setting? Q2-Who treats the POB? Q3-Who believes it is possible to cure occupational burnout, and who believes this is not possible? Q4-Who has the highest proportion of patients who were able to return to work? Q5-Who has the lowest proportion of patients who relapsed? Q6-Does the stage of burnout severity influence the treatment choice? Q7-Does the prognostic belief influence the treatment choice? Q8-Does the proportion of relapsed patients depend on the stage of burnout? Q9-Does the proportion of patients who relapsed is associated with contacts with patient’s employer and health insurance physician or collaborations with other health professionals? Q10-Does the proportion of patients who were able to return to work depend on contacts with patient’s employer and health insurance physician or collaborations with other health professionals?

We considered several datasets for SAP application. A full dataset with data from all respondents was used for the questions 1 and 2. This dataset was then restricted to professionals who treat POBs and split by profession (i.e., physicians and psychologists). These datasets were used for the questions 3 to 10, where the stratification by profession allowed us differentiating treatment options prescribed by physicians and therapies conducted by psychologists in the respective models.

As several questions in the survey allowed an answer option “Impossible to specify” or “I don’t know” and the same participants often chose these options multiple times (indicating the lack of a precise opinion), we excluded these options from the analysis. Keeping them would penalize the statistical performance and result interpretation by increasing the model complexity without adding any meaningful information. Similarly, the respondents with missing values on outcomes or covariates were excluded. The missing values represented less than 15% in average and mostly concerned the open questions rather than questions with multiple choice. The imputation technics in this case have limited or no efficacy.

For each research question we first carried out a univariable logistic or multinomial logistic regression model with the preselected dependent variable and predictor variable. In a second step, multivariable regression models were applied with all the independent variables of interest in the same model. The regression results were reported as Odds Ratio (OR) or Relative Risk Ratio (RRR) with associated 95% confidence interval (IC-95%) and p-value. Data-management procedures and statistical analyses were performed using STATA V 17.0 software.

## Results

### Sample description

The sociodemographic and professional characteristics of responding professionals that treat occupational burnout are summarized in Table 2. The descriptive characteristics of physicians and psychologists are provided in Supplementary material S1 and S2 Tables, respectively. Among the 3216 respondents, 2951 reported having POBs, and 1130, including 713 physicians and 410 psychologists, treat them. Most (n = 894, 79%) work in private practice, are German speaking and have 16.9-year professional experience on average. Among physicians treating POB (S1 Table), there was a similar percentage of men (48%) and women (52%) whereas among psychologists treating POB (S2 Table); there were 83% women. Among physicians who treat POBs, most were either GPs (47%) or PPs (48%), while among psychologists the majority were psychotherapists (82%).

**Table 2:** Descriptive characteristics of the study sample.

Characteristics	N	%
Total number of respondents	3216	
Participants confronted to POB	2951	
Participants treating POB (study sample)	1130	100.0
**Sex**		
Male	414	36.6
Female	716	63.4
**Age group**		
Less than 30 years	17	1.5
30–39 years	169	15.0
40–49 years	306	27.1
50–59 years	335	29.7
60–65 years	157	13.9
More than 65 years	146	12.9
**Language of correspondence**		
French	529	46.8
German	570	50.4
Italian	31	2.7
**Principal Swiss region**		
Lake Geneva region (VD, VS, GE)	409	36.2
Espace Mittelland (BE, FR, SO, NE, JU)	228	20.2
Northwestern Switzerland (BS, BL, AG)	138	12.2
Zürich (ZH)	157	13.9
Eastern Switzerland(GL, SH, AR, AI, SG, GR, TG)	97	8.6
Central Switzerland(LU, UR, SZ, OW, NW, ZG)	70	6.2
Ticino (TI)	31	2.7
**Job category**		
Physician	713	63.1
Psychologist	410	36.3
Occupational Health Nurse	2	0.2
Other	5	0.4
**Principal place of work**		
Private practice	894	79.1
Clinic or private care center	60	5.3
Hospital or public clinic	121	10.7
Public company	13	1.2
Private company	22	2.0
Insurance	3	0.3
Other	17	1.5
	Median	IQR
Number of consultations in the last month	85	50–150
Number of consultations for burnout in the last month	4	2–6
Number of years in practice	15	8–25

### Proportion of consultations for persons with occupational burnout in professionals’ practice

**Fig 1:**
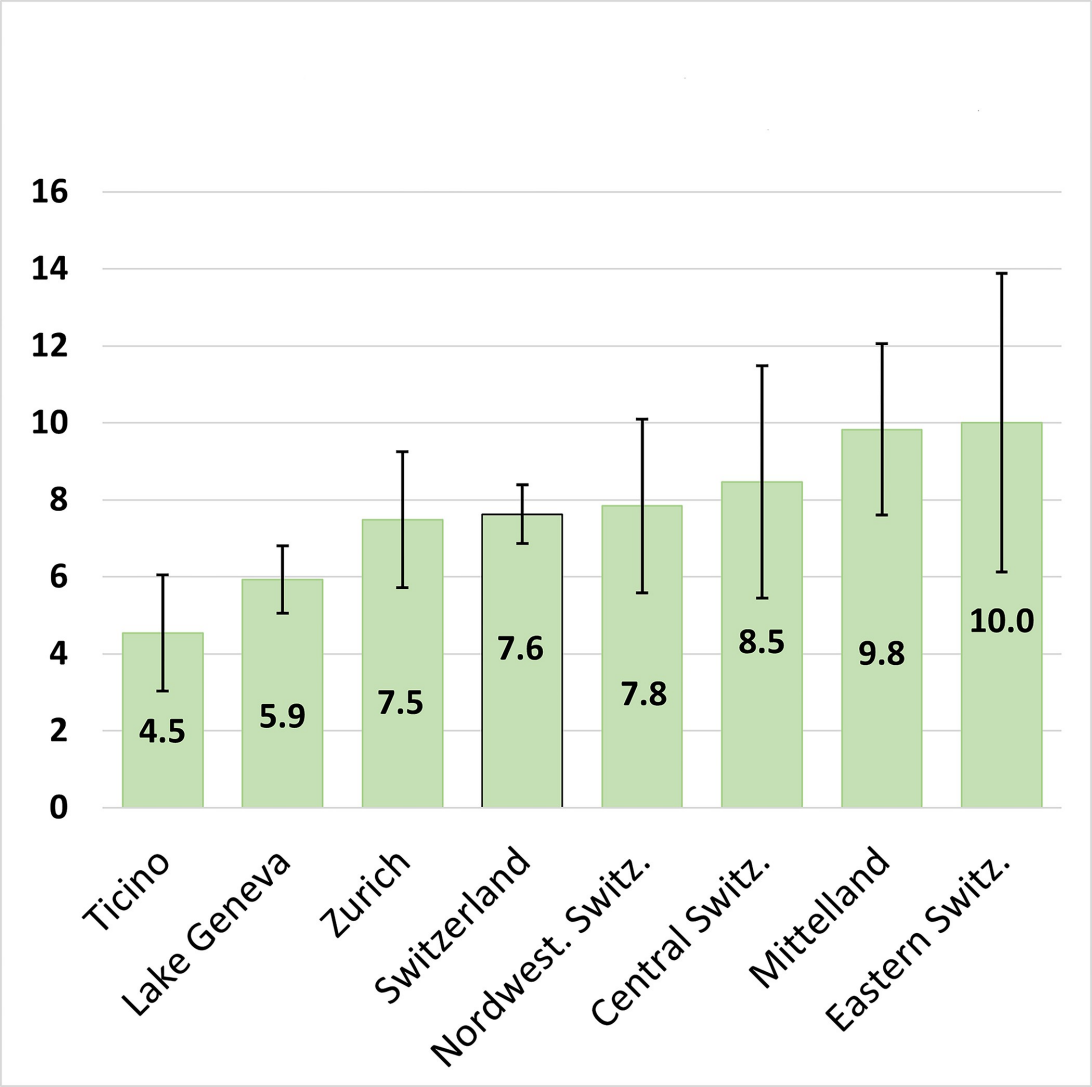
Average proportion of consultations for persons with occupational burnout (mean and 95% confidence interval) in the Swiss health professionals’ activity during the last month (STOBS-VD survey, June 2021).

The proportion of POB consultations was obtained by dividing the reported number of POB consultations by the total number of consultations (assessed over the last month of usual practice by a healthcare professional). This proportion might reflect the volume of outpatient clinical activities dedicated to POB among Swiss health professionals. We observed that this proportion varied according to the profession, medical specialty and psychological specialization [35]. Among physicians, the reported proportion of POB consultations was 6.0%, but those with a double specialty (GP-PPs or OP-GPs) reported the highest proportion (10.8%), followed by OPs (8.7%) and PPs (7.3%). GPs’ reported proportion was 4.8% among their patients. Among psychologists (overall proportion of 8.6%), work psychologists reported the highest proportion (25.6%), while clinical psychologists and psychologists-psychotherapists reported significantly lower proportion of POB among their clients (8.9% and 6.9%, respectively). The reported proportion of POB consultations also differed by professionals’ region of practice (Fig 1).

### Stage of occupational burnout severity and prognostic beliefs

**Fig 2:**
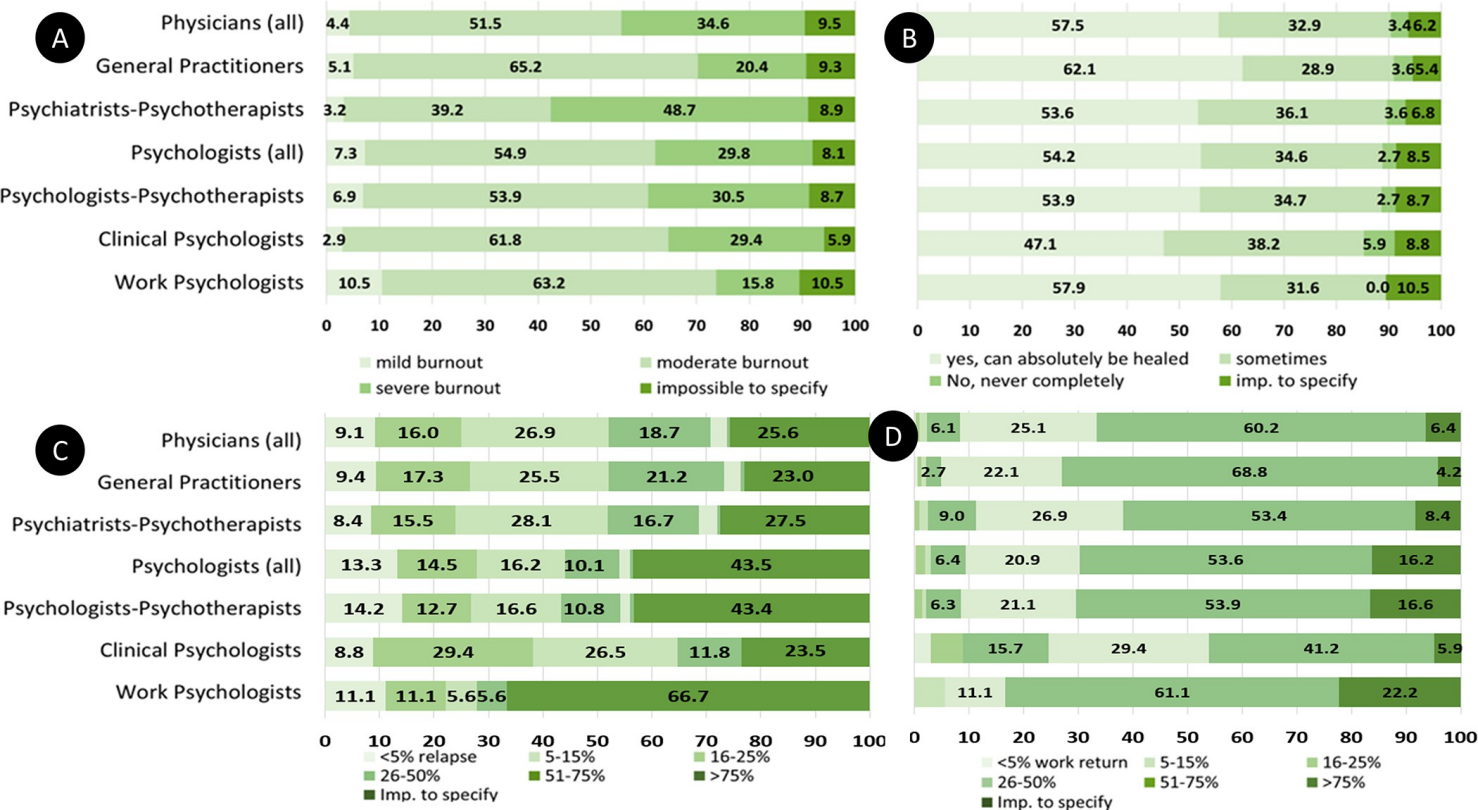
Distribution (in %) of health professionals opting for a certain outcome variant, seen in POB: A–Stage of occupational burnout severity in majority of patients/clients; B–Believes on the prognosis of occupational burnout; C–Proportion of treated patients/clients who relapse; D-Proportion of treated patients/clients who can return to work. *Number of Physicians (all) [713 for A; 711 for B; 706 for C*, *D] / General Practitioners [333 for A; 332 for B; 330 for C*, *D] / Psychiatrists-Psychotherapists [339 for A; 338 for B; 335 for C*, *D] / Psychologists (all) [410 for A*, *B; 407 for C*, *D] / Psychologists-Psychotherapists [334 for A*, *B; 332 for C*, *D] / Clinical Psychologists [34 for A*, *B*, *C*, *D] / Work Psychologists [19 for A*, *B; 18 for C*, *D]*.

Among the major groups of professionals, almost half of the PPs (48.7%) reported that the majority of their POB, had severe burnout while only smaller proportion of GPs (20.4%) and work psychologists (15.8%) reported to have such severe POB (Fig 2, plot A). Psychologists reported more rarely than physicians to have patients that relapse but, in many cases, could not specify it (Fig 2, plot C). In contrast, the proportions physicians reporting that their treated POB could return to work were similar to the proportion of psychologists when omitting those unable to specify this (Fig 2, plot D). As for the prognostic beliefs, the proportion of physicians and psychologists who believe that occupational burnout can absolutely be cured were 54–58%; yet, the proportion of GPs with optimistic prognostic belief was larger (62%) (Fig 2, plot B).

These distributions differed by geographic region, as shown in Supporting information S1 Fig. While the proportion of professionals reporting treating POB was larger in the Eastern Switzerland (10.0) and the Mittelland region (9.8) than in other regions (Fig 1), fewer physicians in these regions reported treating severe POBs (plot C in S1 Fig). The highest proportion of professionals who reported treating severe POB was in Ticino, followed by the Lake Geneva Region (plot C in S1 Fig).

### Professionals’ profiles with respect to occupational burnout treatment and treatment outcomes

The results relative to the research questions 1 to 5 are provided in Supporting information S3–S18 Tables, either for all participants or stratified by profession. Regarding the question Q1, we found that psychologists had a lower probability to be confronted with POBs at the first instance than physicians [OR = 0.55, p = 0.003] (S3 Table). However, psychologists reported to treat POBs more often than physicians [OR = 1.97, p<0.001] (S6 Table), especially psychologists specialized as psychotherapists [OR = 6.35, p<0.001] (S8 Table). Among physicians, psychiatrists reported more frequently than GPs to treat burnout [OR = 12.6, p<0.001] (S7 Table). Since neither age, sex, the number of consultations in the previous month nor the number of years of practice were associated with the treatment of occupational burnout (S6–S8 Tables), no profile emerged as response to the research question Q2.

Regarding the research question Q3, we found that some more pessimistic beliefs were associated with 60+ age in physicians [OR 2.2, p = 0.011] (S9 Table) and female sex in psychologists [OR 2.15, p = 0.024] (S10 Table). German-speaking professionals (both psychologists and physicians) appeared less optimistic regarding burnout prognosis than French speaking professionals, although the regional variation was more pronounced among psychologists than among physicians.

As response to the research question Q4, we found that overall, the proportions of professionals reporting great proportion (over 75%) of their BOP being able to return to work was high (53.9–60.2%, plot D in Fig 2), burnout care being perceived to be quite effective for over ¾ of patients. GPs were the most numerous (68.8%) to report this opinion, while the clinical psychologists the least (41.2%). This was also the case of physicians whose waiting time for consultation appointment is longer than one month (compared to physicians with a sooner consultation possibility) and of physicians practicing in the regions of Zurich, Eastern Switzerland and Ticino (compared to physicians in Lake Geneva region) (S11 Table). In contrast, more physicians practicing in the Central Switzerland region reported that most of their treated patients were able to return to work than physicians in the Geneva Lake region. Among psychologists, we observed no regional variation (S12 Table). Compared to clinical psychologists, psychologists-psychotherapists reported significantly more often a high proportion of clients who returned to work. Psychologists aged between 40 and 59 years old, had also a higher proportion of such clients that younger psychologists.

Regarding the research question Q5, we found that in both physicians and psychologists, 60+ age and female sex were associated with a lower reporting of relapses among the treated POBs. In contrast, a longer waiting time for consultation appointment, was associated with a higher chance to have more relapsed patients after treatment among physicians (S13 Table). Among psychologists, an opposite association was observed (S14 Table).

### Factors associated with the choice of treatment modalities

As expected, the choice of treatment modalities varied across professional specialty or specialization (S2 Fig). The proportion of GPs who report prescribing sick leaves is higher (97.9%) than in PPs (92.3%), while PPs are the most frequent reporters of pharmacological treatment prescription (89.1%, Plot A in S2 Fig). Compared to GPs, a larger proportion of PPs reports to collaborate with pharmacologists (3.5%) and to contact POB’s employer (32.7%) and health insurance (26.0%). Among psychologists, work psychologists differ from other psychologists by reporting more frequently practicing POB (psycho)education and coaching, namely on how to negotiate with employer and family (47.4%), as well as physical exercise (68.4%). The proportion of work psychologists who reported to contact POB’s employer (36.8%) is also the highest compared to other psychologists (Plot B in S2 Fig). Besides profession and specialization, we observed important regional variation in treatment modalities chosen by both physicians and psychologists (S15 and S16 Tables). Physicians in Ticino and in the German-speaking regions were more prone to contact POB’s employer or health insurance doctor in addition to the treatment prescription than in the Lake Geneva region (S15 Table). This was especially the case among physicians with specialty other than GPs.

The probability for physicians to contact POB’s employer as part of the treatment procedure was associated with a more pessimistic belief on burnout prognosis (RRR = 1.78, 95%-CI = 1.20–2.62), but after adjustment for other covariates, the association turned to a borderline statistical significance (RRR = 1.48, 95%-CI = 0.97–2.26) (S15 Table). Similar associations were observed for a combination of the treatment prescription, employer contact and interdisciplinary collaborations, however, it was not associated with practitioner’s belief on burnout prognosis. In psychologists, a pessimistic belief on burnout prognosis was also associated with the combination of therapy and contact with the POB’ employer, with a similar association strength (RRR = 1.81, and 1.63, before and after adjustment, respectively) as among physicians (S16 Table). The combination of therapy with employer and/or health insurance contact and collaboration with other professionals among psychologists was twice more frequent for clients with severe burnout (S17 Table). In physicians, the stage of burnout severity was associated with employer contacts and interdisciplinary collaborations only in univariable analysis.

In response to the research question Q8, we found that the proportion of relapsed patients reported by respondents does not depend on the stage of burnout severity (data not shown). The results corresponding to the research question Q9 and Q10 are provided in the S18 Table. We found no association between interdisciplinary contacts or collaborations and the proportion of relapsed patients reported by healthcare professionals. The OR corresponding to the employer contact by psychologists was 2.40 (p = 0.02) in the univariable model, but dropped to 1.87, statistically nonsignificant in the multivariable model (S18 Table). The association between the collaboration with pharmacologist and proportion of patients who were able to return to work was negative and remained statistically significant after adjustment for cofounders (OR = 0.13 (95%-CI = 0.02–0.66)), suggesting that professionals seek pharmacologist help when their POB cannot manage to return to work.

## Discussion

### Main findings

The study provided a state of the art on the number and distribution of Swiss professionals confronted with POBs, the estimated average volume of consultations dedicated to management of POBs in their practice, the severity of burnout in their POBs, their general beliefs on burnout prognosis, and treatment options chosen including interdisciplinary collaborations and collaboration with POB’s employer and health insurance. The latter was particularly interesting since the Practical recommendations on burnout treatment released by the Swiss network of experts on burnout highlight the importance of interdisciplinarity in the POB treatment [4, 5]. The study showed that POB management constitutes 5 to 25% of health care professionals’ outpatient consultations, with an inequal distribution of POBs across professionals’ specialties and specializations, but also across geographic regions. The latter might raise an issue of health inequality if the Swiss POBs cannot receive the same health care depending on where they live. This raises in turn a crucial issue of perception and measurement of occupational burnout across regions and cultures in Switzerland.

Indeed, regional variation was the most salient and consistent finding in most research questions, especially those aimed to characterize professionals’ attributes (Questions 1–5). The regional variation in practice can originate from several sources including regional differences in professionals’ initial and continuous education; disparities in health organization and healthcare services availability and accessibility; cultural differences in perception of mental illness and occupational burnout as well as their (de)stigmatization; and influence from border countries. The latter was pointed out in health insurance statistics, according to which sick leaves are significantly more frequent and longer in the French-speaking Switzerland [31, 35]. Such regional variations in professionals’ practice are also remarkable since the Practical recommendations on burnout treatment exist in Switzerland since 2016 [4, 5], and probably reflect professionals’ individual sensitivity about the problem and its management.

We observed that the stage of burnout severity determines the treatment choice as does the health care professionals’ beliefs on the burnout prognosis. Physicians and psychologists tend to add more collaborative actions and contacts with POB’s employer and/or health insurance mostly for severe POBs and a pessimistic prognosis. This echoes the professionals’ identified gap in the current practice and the need of tripartite collaboration (POB, employer, health professionals) as early as possible [38–40].

All these results seem logical, however by objectivating them using a large study sample, a comprehensive questionnaire developed by a multidisciplinary research team, and a rigorous analytical scheme, these results are the first to translate empirical guesses on burnout management in Swiss outpatient sector into scientific data [25]. For this, some abstractions were still necessary, namely the outcome variables allowing to compare and to look at the current practices from a care quality and effectiveness perspective. These abstractions deserve a careful definition and interpretation, with consideration of the study context.

### Relevance and accuracy of the studied variables

We defined four outcomes to analyze the professionals’ profiles and practices. The stage of occupational burnout was defined using a set of open questions on how the professional defines the burnout and each of its stages of development/severity, considering three main stages: mild or early burnout, moderate burnout and advanced or clinically severe burnout. The responses were translated and analyzed using the thematic context method in MaxQDA. The resulting definitions were consistent across professions and regions [35], thus we are confident that respondents correctly evaluated this outcome for most of their POBs.

Regarding the belief on the burnout prognosis, an average assessment for most of one’s POBs can be more challenging, as prognosis depends on multiples factors such as the etiology, number and severity of symptoms, therapeutical alliance, patient’s treatment compliance and collaboration, as well as availability of the others’ support and collaboration. We observed, however, that most respondents could assess and report their general prognostic beliefs, and that the proportion of those who could not specify it was even lower that for the stage of burnout (Fig 2).

Conversely, the proportion of relapsed patients among the treated POB raised a concern, since for a quarter of physicians and more than 40% of psychologists, especially the work psychologists, it was impossible to precise the relapse rate (Fig 2). The discussion with the scientific committee helped contextualize it, to understand the reason and the impact on the study results. Indeed, one reason is that a burnout relapse is rarely announced to the professional by the patient. Moreover, in a psychotherapy context, a patient who relapsed may tend to change his/her psychotherapist, either following a misalliance with the therapist or for seeking a new or different treatment approach. Therefore, the accuracy of this outcome may be an issue. Since we excluded those who could not estimate the average rate of relapse in their patents/clients, the remaining subsample is likely to be too small to yield results with sufficient statistical power. This can explain why most associations with this variable are statistically non-significant and challenging to interpret.

Finally, the average rate of POBs ability to return to work after the treatment was well assessed. Return to work generally symbolizes the recovery [41] or a “return to the normality” [42]. Since return to work is likely to be an outcome, seen as the therapy effect, it can also correspond to the end of the treatment when return to work seems successful [43, 44]. Therefore, it is relevant to assess and to examine this outcome in relation with the treatment modalities as we did.

Regarding the treatment options, in this study we focused on a combination of usually prescribed treatments or therapies with the contact of POBs’ employers, health insurance and collaborations with other health professionals. For this, we abstracted a 7-class aggregated variable from 18 options that respondents could select in a multiple-choice question. By doing this we collapsed the details on therapies prescribed and conducted (S2 Fig), as their consideration without individual POB data would be meaningless. In contrast, it would be relevant and interesting to consider the different combinations of treatment options delivered at an individual POB and professional level. This was not possible in the frame of this study but could be feasible in a prospective cohort study of POBs.

### Contextual relevance of study findings

It is noteworthy that this study was initiated just before the Covid-19 pandemic. The survey questionnaire was distributed when the preparation of the 1^st^ vaccination campaign started, which explains that some cantonal associations of GPs and family doctors refused to collaborate and promote this study. This also can explain the relatively low participation rate, although it is comparable to other surveys conducted in the field before or after the pandemic [45]. However, as the Covid-19 pandemic increased the incidence and prevalence of mental ill-health generally and of burnout particularly [28, 46], the proportion of consultations for POB estimated in this study is likely to under-represent the post-pandemic reality. The pandemic might also have increased the proportion of severe POBs and/or OB relapses, and have decreased the proportion of those who are able to return to work, as the work conditions worsened in many instances [6, 47]. Consequently, the study findings reflecting the pre-pandemic situation cannot be directly extrapolated to the pandemic and post-pandemic situation and should be confirmed in an evolved context.

The new national regulation authorizing psychologists-psychotherapists to practice within the framework of compulsory health insurance independently from psychiatrists is another contextual change to mention. This regulation entered in operation in July 2022 and might change the figures on outcome distribution across professions and regions observed in the present study. This could be another reason for repeating this study in a near future and check the result reliability in a new context.

### Study limitations

This study has several limitations that deserve discussion. First, its cross-sectional design is suitable for producing a reliable picture of the health care professionals in outpatient sector dealing with POB and describe their activities in general but precludes a formal comparison of their practices with the Practical recommendations on burnout treatment released by the Swiss network of experts on burnout [4, 5]. Therefore, such a comparison was beyond the scope of the present study but could be valuable in the future. Second, the 19%-overall response-rate was rather modest but that of GPs (12%) was particularly low. Given the Covid-19 pandemic context, such a low participation may conduct to a self-selection of healthcare professionals the most concerned with BOP management. In turn, this may lead to an over-estimation of POB proportion and a biased description of practice distributions. The structural analysis of respondents by comparison with the national distribution of medical demographic data and analysis of partial non-response revealed few deviations from the source population, which was identical to the target population. The proportion of POB consultations corrected for the non-response by applying sampling weights was quite like the unweighted estimate (4.85 instead of 4.47 in GPs; for more detailed method description and results please see the reference [35]). Consequently, we decided to use unweighted data. Although using weighted data for non-response in inferential statistical analyses might help control the non-response bias, there are arguments both for and against this technique [48–50]. As this study was explorative by nature, our focus was on interpreting and communicating results without the added complication of weight management. Third, the proportion of POB in healthcare professional practice may have been calculated differently across study participants. In the questionnaire, we asked how many consultations were performed during the last month and how many of the patients/clients consulted during the last month were experiencing occupational burnout. We did not provide clear instructions that for reporting the latter, it is important to focus on population at risk and to not count consultations for occupationally inactive patients/clients (e.g., retired or students). It is also uncertain how participants counted consultations and patients, especially when answering an online questionnaire. It is likely that they provided their answers using the same units (i.e., either as the number of consultations or the number of consulted patients), however, this does not prevent against possible double countings of patients/clients who have been seen more than once over the past month. Consequently, the estimated proportion of POB consultations in the healthcare practice is more a crude proxy for measuring the volume of clinical activity dedicated to occupational burnout treatment than for measuring the prevalence of POB in the outpatient sector. For calculating the latter, individual patient data collected using a different study design are necessary, as recently shown in patients consulted by German GPs [51].

As we could only assess the outcomes on an aggregated level (i.e., as an average for most professionals’ POBs), some of observed associations can be ecological and need confirmation in future studies based on individuals-level data. As far as we know, such studies of POB do not exist in Switzerland and are also extremely seldom abroad [52–54]. Such studies will also enable comparing the effectiveness of different treatment options and their combination and producing or updating practice recommendations regarding burnout management.

Although we limited the number of statistical analyses, multiple comparisons using the same dataset may be still an issue, leading to an increased likelihood of finding significant results by chance. Yet, the result interpretation and validation were conducted in collaboration with the project scientific committee members to stay true to the original research questions and hypotheses and enhance the validity and credibility of the study.

### Further perspectives

In light with the above-mentioned study limitations and contextual particularities, this study provided several insights that might be helpful for the study replication or for the future efforts. Regarding a better sampling scheme, a probabilistic sampling of healthcare professionals could be more appropriate to the phenomenon of occupational burnout and to the structure of the Swiss outpatient sector. Regarding the questionnaire, we believe that it could be improved by using a more precise definition of some variables and clear instructions on how the variables should be measures (e.g., count of consultations *versus* count of patients; last month *versus* last month of usual practice i.e., beyond the pandemic context). The future studies might use additional research instruments to better capture the sequence of services per case and possible incorporate some qualitative techniques that make more nuanced use of the interaction between authors and professionals or provide comparison with expert recommendations. For this the future research should adhere to a formal evaluative framework, with appropriated study designs and theoretical models. More precisely, our findings suggest the relevance and interest of using the Service needs coverage model [55] to evaluate the extent to which existing resources in the outpatient sector effectively meet the needs of the POB. This model is useful for identifying gaps in service provision and strategies to improve coverage and accessibility of the services from the POB perspective [56]. Alternatively, the Model of access to care allows evaluating the availability, accessibility, accommodation, affordability and accessibility of the healthcare for POB [57]. Furthermore, the Care integration model can be useful for assessing gaps in care, or poor care coordination leading to an adverse impact on care experiences and care outcomes [58]. Similarly, the Care coordination model [59] can usefully fit the issues related to the interdisciplinary collaboration and interaction with POB employer and health insurer, identified by the study participants. Dimensions such as healthcare effectiveness, safety, patient-centeredness, timeliness, efficiency, and equity can be thoroughly assessed using the Donabedian’s model of quality of care [60, 61], the Model of effectiveness of care [62, 63], or the Systems Engineering Initiative for Patient Safety model [64]. Finally, the Knowledge-attitude-behavior model [65] and Knowledge, attitude, and practice surveys [66] can help thoroughly assess what is known (knowledge), believed (attitude), and done (practiced) by the health professionals treating the POB.

## Conclusion

This descriptive study is the first to objectify the importance of occupational burnout as a health care need in the outpatient sector in Switzerland. In particular, the study findings point out its relative importance in general practitioner practice and a potential interest of their training as initiators and coordinators of burnout treatment. The study suggests that most professionals collaborate with other health professionals and patient’s employer and health insurance only in cases of severe burnout, pessimistic beliefs on burnout prognosis, and when the return to work is challenging. The support of this collaborative, interdisciplinary efforts and their extension to earlier burnout stage might thus improve the treatment outcomes. Education regarding occupational burnout detection and management seems also necessary, especially in GPs pre-graduate curriculum. As the study had an exploratory nature using a cross-sectional survey design and aggregated outcomes, its findings should be considered as first descriptive data, motivating further research. The future research should adhere to a formal evaluative framework, with appropriated theoretical models and study designs using individual POB data.

## Supporting information

S1 Table Descriptive characteristics of the physician subsample. (DOCX)

S2 Table Descriptive characteristics of the psychologist subsample. (DOCX)

S3 Table Attributes of Swiss health professionals confronted to burned-out patients (n = 3088). (DOCX)

S4 Table Attributes of Swiss physicians confronted to burned-out patients (n = 1649). (DOCX)

S5 Table Attributes of Swiss psychologists confronted to burnout clients (n = 1274). (DOCX)

S6 Table Attributes of Swiss health professionals who treat burned-out patients (n = 1554). (DOCX)

S7 Table Attributes of physicians who treat burned-out patients (n = 990). (DOCX)

S8 Table Attributes of Swiss psychologists who treat burnout clients (n = 508). (DOCX)

S9 Table Physicians’ characteristics associated with their beliefs on the burnout prognosis (n = 630). (DOCX)

S10 Table Psychologists’ characteristics associated with their beliefs on the burnout prognosis (n = 329). (DOCX)

S11 Table Physicians’ personnel and professional attributes associated with the highest reported proportion of return to work among their patients treated for burnout (n = 632). (DOCX)

S12 Table Psychologists’ personnel and professional attributes associated with the highest reported proportion of return to work among their patients treated for burnout (n = 306). (DOCX)

S13 Table Physicians’ personnel and professional attributes associated with high (more than 25%) reported proportion of relapsed patients (n = 504). (DOCX)

S14 Table Psychologist’ personnel and professional attributes associated with high (more than 25%) reported proportion of relapsed patients (n = 203). (DOCX)

S15 Table Physicians’ characteristics and beliefs on the prognosis of burnout associated with reported burnout treatment modalities (n = 665). (DOCX)

S16 Table Psychologists’ characteristics and beliefs on the prognosis of burnout associated with reported burnout treatment modalities (n = 359). (DOCX)

S17 Table Associations of caregivers’ specialty / specialization and choice of the treatment options with reporting of severe burnout among their patients / clients. (DOCX)

S18 Table Association of interprofessional contacts and collaborations with reported proportions of return to work and relapses among patients/clients treated for burnout. (DOCX)

S1 Fig Regional variation in the distribution of studies outcomes reported by health professionals who treat burned-out patients / clients (%). (DOCX)

S2 Fig Distribution (in %) of burnout treatment options by Swiss health professionals. (DOCX)
