# Supplementary material for: Diagnosis and treatment of occupational burnout in the Swiss outpatient sector: A national survey of healthcare professionals’ attributes and attitudes
Source: PLoS One. 2024 Dec 11;19(12):e0294834. doi: 10.1371/journal.pone.0294834 (PMC11633953; doi:10.1371/journal.pone.0294834)
Supplement: S1 Table — (DOCX) [file pone.0294834.s001.docx]

S1 Table. Descriptive characteristics of the physician subsample

|  | **N** | **%** |
| --- | --- | --- |
| Physicians all | 1723 |  |
| Physicians confronted to burnout | 1657 |  |
| **Physicians treating burnout patients** | **713** | **100.0** |
| **Sex** |  |  |
| Male | 342 | 48.0 |
| Female | 371 | 52.0 |
| **Age group** |  |  |
| Less than 30 years | 7 | 1.0 |
| 30 - 39 years | 77 | 10.8 |
| 40 - 49 years | 187 | 26.2 |
| 50 - 59 years | 221 | 31.0 |
| 60 - 65 years | 114 | 16.0 |
| More than 65 years | 107 | 15.0 |
| **Language of correspondence** |  |  |
| French | 376 | 52.7 |
| German | 319 | 44.7 |
| Italian | 18 | 2.5 |
| **Principal Swiss region** |  |  |
| Lake Geneva region (VD, VS, GE) | 301 | 42.2 |
| Espace Mittelland (BE, FR, SO, NE, JU) | 132 | 18.5 |
| Northwestern Switzerland (BS, BL, AG) | 85 | 11.9 |
| Zürich (ZH) | 74 | 10.4 |
| Eastern Switzerland (GL, SH, AR, AI, SG, GR, TG) | 69 | 9.7 |
| Central Switzerland (LU, UR, SZ, OW, NW, ZG) | 35 | 4.9 |
| Ticino (TI) | 17 | 2.4 |
| **Specialty** |  |  |
| General physician | 333 | 46.7 |
| Psychiatrist-psychotherapist | 339 | 47.6 |
| Occupational physician | 3 | 0.4 |
| Dual specialty: General medicine and occupational medicine | 8 | 1.1 |
| Dual specialty: General medicine and psychiatry | 13 | 1.8 |
| Dual specialty: Psychiatry and occupational medicine | 1 | 0.1 |
| Other | 16 | 2.2 |
| **Principal place of work** |  |  |
| Private practice | 593 | 83.2 |
| Clinic or private care center | 26 | 3.7 |
| Hospital or public clinic | 73 | 10.2 |
| Public company | 5 | 0.7 |
| Private company | 8 | 1.1 |
| Insurance | 2 | 0.3 |
| Other | 6 | 0.8 |
| **Professional characteristics** | **Mean** | **SD** |
| Number of consultations in the last month | 161.7 | 143.8 |
| Number of consultations for burnout in the last month | 6.4 | 22.4 |
| Number of years in practice | 17.8 | 11.1 |
