# Supplementary material for: Diagnosis and treatment of occupational burnout in the Swiss outpatient sector: A national survey of healthcare professionals’ attributes and attitudes
Source: PLoS One. 2024 Dec 11;19(12):e0294834. doi: 10.1371/journal.pone.0294834 (PMC11633953; doi:10.1371/journal.pone.0294834)
Supplement: S2 Table — (DOCX) [file pone.0294834.s002.docx]

S2 Table. Descriptive characteristics of the psychologist subsample

|  | **N** | **%** |
| --- | --- | --- |
| Psychologists all | 1326 |  |
| Psychologists confronted to burnout | 1176 |  |
| **Psychologists treating burnout clients** | **410** | **100.0** |
| **Sex** |  |  |
| Male | 71 | 17.3 |
| Female | 339 | 82.7 |
| **Age group** |  |  |
| Less than 30 years | 10 | 2.4 |
| 30 - 39 years | 89 | 21.7 |
| 40 - 49 years | 118 | 28.8 |
| 50 - 59 years | 111 | 27.1 |
| 60 - 65 years | 43 | 10.5 |
| More than 65 years | 39 | 9.5 |
| **Language of correspondence** |  |  |
| French | 147 | 35.9 |
| German | 250 | 61.0 |
| Italian | 13 | 3.2 |
| **Principal Swiss region** |  |  |
| Lake Geneva region (VD, VS, GE) | 104 | 25.4 |
| Espace Mittelland (BE, FR, SO, NE, JU) | 95 | 23.2 |
| Northwestern Switzerland (BS, BL, AG) | 52 | 12.7 |
| Zürich (ZH) | 83 | 20.2 |
| Eastern Switzerland (GL, SH, AR, AI, SG, GR, TG) | 27 | 6.6 |
| Central Switzerland (LU, UR, SZ, OW, NW, ZG) | 35 | 8.5 |
| Ticino (TI) | 14 | 3.4 |
| **Specialization** |  |  |
| Clinical psychologist | 34 | 8.3 |
| Occupational Psychologist | 19 | 4.6 |
| Cognitive Psychologist | 3 | 0.7 |
| Social Psychologist | 1 | 0.2 |
| Psychologist-Psychotherapist | 334 | 81.5 |
| Other | 13 | 3.2 |
| Missing | 6 | 1.5 |
| **Principal place of work** |  |  |
| Private practice | 299 | 72.9 |
| Clinic or private care center | 33 | 8.1 |
| Hospital or public clinic | 48 | 11.7 |
| Public company | 6 | 1.5 |
| Private company | 12 | 2.9 |
| Insurance | 1 | 0.2 |
| Other | 11 | 2.7 |
| **Professional characteristics** | **Mean** | **SD** |
| Number of consultations in the last month | 66.1 | 46.2 |
| Number of consultations for burnout in the last month | 4.6 | 6.2 |
| Number of years in practice | 15.3 | 9.7 |
