# Supplementary material for: Diagnosis and treatment of occupational burnout in the Swiss outpatient sector: A national survey of healthcare professionals’ attributes and attitudes
Source: PLoS One. 2024 Dec 11;19(12):e0294834. doi: 10.1371/journal.pone.0294834 (PMC11633953; doi:10.1371/journal.pone.0294834)
Supplement: S3 Table — (DOCX) [file pone.0294834.s003.docx]

S3 Table. Attributes of Swiss health professionals confronted to burned-out patients (n=3088)

|  | **Univariate model^1^** | | **Multivariate model^2^** | |
| --- | --- | --- | --- | --- |
| **Independent variables** | **OR [95% CI]** | **p-value** | **OR [95% CI]** | **p-value** |
| **Age group** |  |  |  |  |
| Less than 30 years | 1.00 | Ref | 1.00 | Ref |
| 30 - 39 years | 2.77 [1.45 - 5.29] | 0.002 | 1.62 [0.80 - 3.30] | 0.181 |
| 40 - 49 years | 4.00 [2.12 - 7.57] | <0.001 | 1.89 [0.90 - 3.94] | 0.091 |
| 50 - 59 years | 5.49 [2.88 - 10.47] | <0.001 | 1.83 [0.83 - 4.05] | 0.134 |
| 60 - 65 years | 9.59 [4.34 - 21.19] | <0.001 | 2.12 [0.79 - 5.69] | 0.134 |
| More than 65 years | 10.31 [4.54 - 23.44] | <0.001 | 2.01 [0.65 - 6.20] | 0.226 |
| **Sex** |  |  |  |  |
| Male | 1.00 | Ref | 1.00 | Ref |
| Female | 0.55 [0.40 - 0.76] | <0.001 | 0.90 [0.61 - 1.32] | 0.587 |
| **Job category** |  |  |  |  |
| Physician | 1.00 | Ref | 1.00 | Ref |
| Psychologist | 0.27 [0.19 - 0.37] | <0.001 | 0.55 [0.38 - 0.81] | 0.003 |
| Occupational Nurse | 0.40 [0.12 - 1.33] | 0.135 | 1.61 [0.36 - 7.17] | 0.535 |
| Other | 0.16 [0.09 - 0.30] | <0.001 | 0.70 [0.34 - 1.46] | 0.340 |
| **Principal place of work** |  |  |  |  |
| Private practice | 1.00 | Ref | 1.00 | Ref |
| Clinic or private care center | 0.39 [0.22 - 0.66] | 0.001 | 0.62 [0.34 - 1.13] | 0.121 |
| Hospital or public clinic | 0.25 [0.17 - 0.36] | <0.001 | 0.37 [0.24 - 0.56] | <0.001 |
| Public company | 0.19 [0.11 - 0.32] | <0.001 | 0.33 [0.18 - 0.59] | <0.001 |
| Private company | 0.22 [0.12 - 0.39] | <0.001 | 0.33 [0.18 - 0.64] | 0.001 |
| Insurance | 0.46 [0.06 - 3.57] | 0.460 | 0.59 [0.07 - 4.69] | 0.619 |
| Other | 0.07 [0.04 - 0.10] | <0.001 | 0.11 [0.07 - 0.18] | <0.001 |
| **Job duration** | 1.04 [1.03 - 1.06] | <0.001 | 1.03 [1.01 - 1.05] | 0.012 |
| **No of consultations** | 1.01 [1.01 - 1.01] | <0.001 | 1.00 [1.00 - 1.01] | 0.004 |

^1^-Logistic regression model with confrontation to burnout (yes/no, Reference: yes) as dependent variable; ^2^-Logistic regression model with confrontation to burnout as dependent variable, adjusted for all co-variables examined in the univariate analysis
