# Supplementary material for: Diagnosis and treatment of occupational burnout in the Swiss outpatient sector: A national survey of healthcare professionals’ attributes and attitudes
Source: PLoS One. 2024 Dec 11;19(12):e0294834. doi: 10.1371/journal.pone.0294834 (PMC11633953; doi:10.1371/journal.pone.0294834)
Supplement: S4 Table — (DOCX) [file pone.0294834.s004.docx]

S4 Table. Attributes of Swiss physicians confronted to burned-out patients (n= 1649)

|  | **Univariate model^1^** | | **Multivariate model^2^** | | |
| --- | --- | --- | --- | --- | --- |
| **Independent variables** | **OR [95% CI]** | **p-value** |  | **OR [95% CI]** | **p-value** |
| **Age group** |  |  |  |  |  |
| Less than 30 years | 1.00 | Ref |  | 1.00 | Ref |
| 30 - 39 years | 4.38 [0.82 - 23.42] | 0.084 |  | 1.90 [0.33 - 10.93] | 0.472 |
| 40 - 49 years | 3.73 [0.78 - 17.85] | 0.100 |  | 1.63 [0.28 - 9.37] | 0.584 |
| 50 - 59 years | 5.90 [1.21 - 28.71] | 0.028 |  | 1.89 [0.29 - 12.18] | 0.504 |
| 60 - 65 years | 10.62 [1.77 - 63.85] | 0.010 |  | 2.35 [0.27 - 20.37] | 0.437 |
| More than 65 years | 5.21 [1.00 - 27.23] | 0.051 |  | 0.91 [0.10 - 7.88] | 0.928 |
| **Sex** |  |  |  |  |  |
| Male | 1.00 | Ref |  | 1.00 | Ref |
| Female | 1.28 [0.74 - 2.18] | 0.375 |  | 1.29 [0.71 - 2.36] | 0.400 |
| **Specialty*** |  |  |  |  |  |
| General physician | 1.00 | Ref |  | 1.00 | Ref |
| Psychiatrist | 0.75 [0.39 - 1.45] | 0.395 |  | 0.97 [0.45 - 2.07] | 0.931 |
| Occupational physician | 0.12 [0.04 - 0.31] | <0.001 |  | 0.37 [0.10 - 1.38] | 0.140 |
| General Physician and Psychiatrist | 0.49 [0.06 - 3.81] | 0.494 |  | 0.61 [0.07 - 5.14] | 0.652 |
| Other | 0.16 [0.07 - 0.34] | <0.001 |  | 0.20 [0.09 - 0.48] | <0.001 |
| **Principal place of work** |  |  |  |  |  |
| Private practice |  |  |  |  |  |
| Clinic or private care center | 0.22 [0.09 - 0.57] | 0.002 |  | 0.22 [0.08 - 0.57] | 0.002 |
| Hospital or public clinic | 0.16 [0.09 - 0.32] | <0.001 |  | 0.26 [0.12 - 0.58] | 0.001 |
| Public company | 0.12 [0.03 - 0.43] | 0.001 |  | 0.24 [0.04 - 1.38] | 0.111 |
| Private company | 0.16 [0.05 - 0.57] | 0.004 |  | 0.18 [0.04 - 0.78] | 0.022 |
| Insurance | 0.12 [0.01 - 1.03] | 0.054 |  | 0.17 [0.02 - 1.91] | 0.151 |
| Other | 0.08 [0.03 - 0.26] | <0.001 |  | 0.13 [0.04 - 0.47] | 0.002 |
| **Job duration** | 1.02 [1.00 - 1.05] | 0.096 |  | 1.03 [0.99 - 1.07] | 0.180 |
| **No of consultations** | 1.00 [1.00 - 1.01] | 0.009 |  | 1.00 [1.00 - 1.00] | 0.225 |

^1^-Logistic regression model with confrontation to burnout (yes/no, Reference: yes) as dependent variable; ^2^-Logistic regression model with confrontation to burnout as dependent variable, adjusted for all co-variables examined in the univariate analysis; * the categories "General and Occupational Physician" and "Psychiatrist and Occupational Physician" were omitted because of too few observed numbers
