# Supplementary material for: Diagnosis and treatment of occupational burnout in the Swiss outpatient sector: A national survey of healthcare professionals’ attributes and attitudes
Source: PLoS One. 2024 Dec 11;19(12):e0294834. doi: 10.1371/journal.pone.0294834 (PMC11633953; doi:10.1371/journal.pone.0294834)
Supplement: S5 Table — (DOCX) [file pone.0294834.s005.docx]

S5 Table. Attributes of Swiss psychologists confronted to burnout clients (n=1274)

|  | **Univariate model^1^** | | **Multivariate model^2^** | | |
| --- | --- | --- | --- | --- | --- |
| **Independent variables** | **OR [95% CI]** | **p-value** |  | **OR [95% CI]** | **p-value** |
| **Age group** |  |  |  |  |  |
| Less than 30 years | 1.00 | Ref |  | 1.00 | Ref |
| 30 - 39 years | 1.65 [0.71 - 3.85] | 0.248 |  | 1.31 [0.49 - 3.56] | 0.590 |
| 40 - 49 years | 2.28 [0.97 - 5.35] | 0.059 |  | 1.68 [0.59 - 4.77] | 0.333 |
| 50 - 59 years | 2.93 [1.23 - 6.98] | 0.015 |  | 1.57 [0.51 - 4.87] | 0.435 |
| 60 - 65 years | 4.34 [1.53 - 12.30] | 0.006 |  | 1.82 [0.45 - 7.36] | 0.402 |
| More than 65 years | 11.59 [2.88 - 46.70] | 0.001 |  | 13.87 [1.28 - 150.42] | 0.031 |
| **Sex** |  |  |  |  |  |
| Male | 1.00 | Ref |  | 1.00 | Ref |
| Female | 0.63 [0.37 - 1.07] | 0.090 |  | 0.65 [0.34 - 1.22] | 0.176 |
| **Specialization** |  |  |  |  |  |
| Occupational psychologist | 1.00 | Ref |  | 1.00 | Ref |
| Clinical psychologist | 0.45 [0.17 - 1.16] | 0.100 |  | 0.22 [0.07 - 0.67] | 0.008 |
| Cognitive psychologist | 0.13 [0.04 - 0.40] | <0.001 |  | 0.06 [0.02 - 0.23] | <0.001 |
| Social psychologist | 0.50 [0.05 - 4.86] | 0.550 |  | 0.24 [0.02 - 2.75] | 0.253 |
| Psychologist-Psychotherapist | 0.87 [0.37 - 2.05] | 0.746 |  | 0.30 [0.10 - 0.88] | 0.028 |
| Other | 0.33 [0.13 - 0.85] | 0.021 |  | 0.34 [0.11 - 1.03] | 0.057 |
| **Principal place of work *** |  |  |  |  |  |
| Private practice | 1.00 | Ref |  | 1.00 | Ref |
| Clinic or private care center | 0.80 [0.38 - 1.68] | 0.560 |  | 1.38 [0.59 - 3.24] | 0.462 |
| Hospital or public clinic | 0.35 [0.21 - 0.58] | <0.001 |  | 0.56 [0.32 - 0.98] | 0.042 |
| Public company | 0.28 [0.15 - 0.51] | <0.001 |  | 0.34 [0.17 - 0.70] | 0.003 |
| Private company | 0.33 [0.15 - 0.72] | 0.005 |  | 0.31 [0.12 - 0.84] | 0.022 |
| Other | 0.11 [0.06 - 0.17] | <0.001 |  | 0.13 [0.07 - 0.23] | <0.001 |
| **Job duration** | 1.05 [1.03 - 1.07] | <0.001 |  | 1.03 [0.99 - 1.06] | 0.111 |
| **No of consultations** | 1.02 [1.01 - 1.02] | <0.001 |  | 1.01 [1.01 - 1.02] | <0.001 |

^1^-Logistic regression model with confrontation to burnout (yes/no, Reference: yes) as dependent variable; ^2^-Logistic regression model with confrontation to burnout as dependent variable, adjusted for all co-variables examined in the univariate analysis; * The category "Insurance" was omitted because of small observation numbers
