# Supplementary material for: Diagnosis and treatment of occupational burnout in the Swiss outpatient sector: A national survey of healthcare professionals’ attributes and attitudes
Source: PLoS One. 2024 Dec 11;19(12):e0294834. doi: 10.1371/journal.pone.0294834 (PMC11633953; doi:10.1371/journal.pone.0294834)
Supplement: S6 Table — (DOCX) [file pone.0294834.s006.docx]

S6 Table. Attributes of Swiss health professionals who treat burned-out patients (n=1554)

|  | **Univariate model^1^** | |  | **Multivariate model^2^** | |
| --- | --- | --- | --- | --- | --- |
| **Independent variables** | **OR [95% CI]** | **p** |  | **OR [95% CI]** | **p** |
| **Age group** |  |  |  |  |  |
| Less than 30 years | 1.00 | Ref |  | 1.00 | Ref |
| 30 - 39 years | 0.47 [0.15 - 1.43] | 0.184 |  | 0.33 [0.10 - 1.08] | 0.066 |
| 40 - 49 years | 0.56 [0.18 - 1.69] | 0.302 |  | 0.46 [0.14 - 1.53] | 0.207 |
| 50 - 59 years | 0.62 [0.20 - 1.86] | 0.391 |  | 0.59 [0.17 - 2.00] | 0.394 |
| 60 - 65 years | 0.79 [0.25 - 2.45] | 0.678 |  | 0.72 [0.20 - 2.64] | 0.623 |
| More than 65 years | 1.11 [0.35 - 3.52] | 0.862 |  | 0.93 [0.24 - 3.63] | 0.914 |
| **Sex** |  |  |  |  |  |
| Male | 1.00 | Ref |  | 1.00 | Ref |
| Female | 1.16 [0.92 - 1.46] | 0.210 |  | 1.08 [0.83 - 1.41] | 0.555 |
| **Job category** |  |  |  |  |  |
| Physician | 1.00 | Ref |  | 1.00 | Ref |
| Psychologist | 1.56 [1.21 - 2.01] | 0.001 |  | 1.97 [1.44 - 2.70] | <0.001 |
| Occupational Nurse | 0.05 [0.01 - 0.24] | <0.001 |  | 0.11 [0.02 - 0.54] | 0.006 |
| Other | 0.19 [0.06 - 0.54] | 0.002 |  | 0.37 [0.12 - 1.18] | 0.093 |
| **Principal place of work** |  |  |  |  |  |
| Private practice | 1.00 | Ref |  | 1.00 | Ref |
| Clinic or private care center | 0.52 [0.33 - 0.82] | 0.005 |  | 0.49 [0.31 - 0.80] | 0.004 |
| Hospital or public clinic | 0.56 [0.40 - 0.79] | 0.001 |  | 0.55 [0.38 - 0.80] | 0.002 |
| Public company | 0.11 [0.06 - 0.20] | <0.001 |  | 0.08 [0.04 - 0.17] | <0.001 |
| Private company | 0.24 [0.14 - 0.44] | <0.001 |  | 0.21 [0.11 - 0.41] | <0.001 |
| Insurance | 0.21 [0.05 - 0.94] | 0.041 |  | 0.18 [0.04 - 0.83] | 0.028 |
| Other | 0.22 [0.12 - 0.43] | <0.001 |  | 0.14 [0.07 - 0.28] | <0.001 |
| **Job duration** | 1.01 [1.00 - 1.02] | 0.130 |  | 0.99 [0.97 - 1.01] | 0.344 |
| **No of consultations** | 1.00 [1.00 - 1.00] | 0.004 |  | 1.00 [1.00 - 1.00] | <0.001 |

1-Logistic regression model with treatment of burnout (Cat: yes/no, Reference: yes) as dependent variable; 2-Logistic regression model with treatment of burnout as dependent variable, adjusted for all co-variables examined in the univariate analysis
