# Supplementary material for: Diagnosis and treatment of occupational burnout in the Swiss outpatient sector: A national survey of healthcare professionals’ attributes and attitudes
Source: PLoS One. 2024 Dec 11;19(12):e0294834. doi: 10.1371/journal.pone.0294834 (PMC11633953; doi:10.1371/journal.pone.0294834)
Supplement: S7 Table — (DOCX) [file pone.0294834.s007.docx]

S7 Table. Attributes of physicians who treat burned-out patients (n=990)

|  | **Univariate model^1^** | |  | **Multivariate model^2^** | |
| --- | --- | --- | --- | --- | --- |
| **Independent variables** | **OR [95% CI]** | **p** |  | **OR [95% CI]** | **p** |
| **Age group** |  |  |  |  |  |
| Less than 30 years | 1.00 | Ref |  | 1.00 | Ref |
| 30 - 39 years | 0.23 [0.03 - 1.92] | 0.174 |  | 0.12 [0.01 - 1.21] | 0.072 |
| 40 - 49 years | 0.30 [0.04 - 2.51] | 0.268 |  | 0.14 [0.01 - 1.42] | 0.096 |
| 50 - 59 years | 0.34 [0.04 - 2.80] | 0.315 |  | 0.12 [0.01 - 1.26] | 0.077 |
| 60 - 65 years | 0.44 [0.05 - 3.70] | 0.450 |  | 0.13 [0.01 - 1.42] | 0.093 |
| More than 65 years | 0.64 [0.07 - 5.42] | 0.680 |  | 0.14 [0.01 - 1.71] | 0.124 |
| **Sex** |  |  |  |  |  |
| Male | 1.00 | Ref |  | 1.00 | Ref |
| Female | 0.99 [0.76 - 1.30] | 0.954 |  | 1.12 [0.81 - 1.55] | 0.508 |
| **Specialty*** |  |  |  |  |  |
| General physician | 1.00 | Ref |  | 1.00 | Ref |
| Psychiatrist | 10.27 [6.46 - 16.33] | <0.001 |  | 12.63 [7.51 - 21.24] | <0.001 |
| Occupational physician | 0.10 [0.03 - 0.34] | <0.001 |  | 0.19 [0.04 - 0.77] | 0.020 |
| General and Occupational physician | 1.33 [0.40 - 4.48] | 0.642 |  | 2.38 [0.58 - 9.79] | 0.228 |
| Other | 0.46 [0.24 - 0.90] | 0.023 |  | 0.45 [0.22 - 0.89] | 0.023 |
| **Principal place of work** |  |  |  |  |  |
| Private practice | 1.00 | Ref |  | 1.00 | Ref |
| Clinic or private care center | 0.47 [0.26 - 0.86] | 0.014 |  | 0.49 [0.25 - 0.96] | 0.039 |
| Hospital or public clinic | 0.73 [0.48 - 1.12] | 0.153 |  | 0.40 [0.22 - 0.72] | 0.002 |
| Public company | 0.18 [0.06 - 0.53] | 0.002 |  | 0.29 [0.07 - 1.25] | 0.097 |
| Private company | 0.58 [0.19 - 1.78] | 0.340 |  | 1.43 [0.34 - 6.12] | 0.627 |
| Insurance | 0.18 [0.03 - 0.99] | 0.049 |  | 0.10 [0.01 - 1.64] | 0.107 |
| Other | 1.08 [0.22 - 5.40] | 0.923 |  | 1.55 [0.29 - 8.29] | 0.606 |
| **Job duration** | 1.01 [1.00 - 1.03] | 0.033 |  | 1.01 [0.99 - 1.04] | 0.224 |
| **No of consultations** | 1.00 [1.00 - 1.00] | 0.001 |  | 1.00 [1.00 - 1.00] | 0.723 |

1-Logistic regression model with treatment of burnout (Cat: yes/no, Reference: yes) as dependent variable; 2-Logistic regression model with treatment of burnout as dependent variable, adjusted for all co-variables examined in the univariate analysis; * the categories "General Physician and Psychiatrist" and "Psychiatrist and Occupational Physician" were omitted because of small observation number
