# Supplementary material for: Diagnosis and treatment of occupational burnout in the Swiss outpatient sector: A national survey of healthcare professionals’ attributes and attitudes
Source: PLoS One. 2024 Dec 11;19(12):e0294834. doi: 10.1371/journal.pone.0294834 (PMC11633953; doi:10.1371/journal.pone.0294834)
Supplement: S8 Table — (DOCX) [file pone.0294834.s008.docx]

S8 Table. Attributes of Swiss psychologists who treat burnout clients (n=508)

|  | **Univariate model^1^** | |  | **Multivariate model^2^** | |
| --- | --- | --- | --- | --- | --- |
| **Independent variables** | **OR [95% CI]** | **p-value** |  | **OR [95% CI]** | **p-value** |
| **Age group** |  |  |  |  |  |
| Less than 30 years | 1.00 | Ref |  | 1.00 | Ref |
| 30 - 39 years | 0.52 [0.11 - 2.51] | 0.419 |  | 0.33 [0.05 - 2.25] | 0.258 |
| 40 - 49 years | 0.74 [0.15 - 3.54] | 0.703 |  | 0.63 [0.09 - 4.49] | 0.648 |
| 50 - 59 years | 0.97 [0.20 - 4.70] | 0.965 |  | 0.84 [0.11 - 6.54] | 0.871 |
| 60 - 65 years | 0.96 [0.18 - 5.13] | 0.958 |  | 0.38 [0.04 - 3.74] | 0.409 |
| More than 65 years | 1.11 [0.20 - 6.21] | 0.902 |  | 0.59 [0.05 - 6.70] | 0.671 |
| **Sex** |  |  |  |  |  |
| Male | 1.00 | Ref |  | 1.00 | Ref |
| Female | 1.31 [0.77 - 2.22] | 0.319 |  | 1.50 [0.76 - 2.95] | 0.244 |
| **Specialization*** |  |  |  |  |  |
| Occupational psychologist | 1.00 | Ref |  | 1.00 | Ref |
| Clinical psychologist | 4.65 [1.85 - 11.68] | 0.001 |  | 2.89 [0.87 - 9.60] | 0.083 |
| Cognitive psychologist | 0.68 [0.15 - 3.09] | 0.622 |  | 0.32 [0.05 - 1.83] | 0.198 |
| Psychologist-Psychotherapist | 11.72 [5.95 - 23.09] | <0.001 |  | 6.35 [2.26 - 17.84] | <0.001 |
| Other | 0.71 [0.29 - 1.74] | 0.456 |  | 0.58 [0.17 - 1.93] | 0.374 |
| **Principal place of work *** |  |  |  |  |  |
| Private practice | 1.00 | Ref |  | 1.00 | Ref |
| Clinic or private care center | 0.39 [0.17 - 0.89] | 0.026 |  | 0.38 [0.15 - 0.99] | 0.047 |
| Hospital or public clinic | 0.30 [0.16 - 0.59] | <0.001 |  | 0.27 [0.13 - 0.57] | 0.001 |
| Public company | 0.03 [0.01 - 0.09] | <0.001 |  | 0.07 [0.02 - 0.24] | <0.001 |
| Private company | 0.10 [0.04 - 0.23] | <0.001 |  | 0.31 [0.09 - 1.04] | 0.057 |
| Other | 0.07 [0.03 - 0.17] | <0.001 |  | 0.10 [0.04 - 0.26] | <0.001 |
| **Job duration** | 1.00 [0.98 - 1.03] | 0.754 |  | 1.00 [0.96 - 1.04] | 0.899 |
| **No of consultations** | 1.02 [1.02 - 1.03] | <0.001 |  | 1.00 [1.00 - 1.01] | 0.268 |

1-Logistic regression model with treatment of burnout (Cat: yes/no, Reference: yes) as dependent variable; 2-Logistic regression model with treatment of burnout as dependent variable, adjusted for all co-variables examined in the univariate analysis; * the categories "Social psychologist" and "Insurance" were omitted because of small observation number
