# Supplementary material for: Diagnosis and treatment of occupational burnout in the Swiss outpatient sector: A national survey of healthcare professionals’ attributes and attitudes
Source: PLoS One. 2024 Dec 11;19(12):e0294834. doi: 10.1371/journal.pone.0294834 (PMC11633953; doi:10.1371/journal.pone.0294834)
Supplement: S9 Table — (DOCX) [file pone.0294834.s009.docx]

S9 Table. Physicians' characteristics associated with their beliefs on the burnout prognosis (n=630)

1-Logistic regression model with prognosis of burnout (can absolutely be cured /can sometimes or never be cured (REFERENCE)) as dependent variable; 2-Logistic regression model with prognosis of burnout as dependent variable, adjusted for all co-variables examined in the univariate analysis
