# Supplementary material for: Diagnosis and treatment of occupational burnout in the Swiss outpatient sector: A national survey of healthcare professionals’ attributes and attitudes
Source: PLoS One. 2024 Dec 11;19(12):e0294834. doi: 10.1371/journal.pone.0294834 (PMC11633953; doi:10.1371/journal.pone.0294834)
Supplement: S11 Table — (DOCX) [file pone.0294834.s011.docx]

S11 Table. Physicians' personnel and professional attributes associated with the highest reported proportion of return to work among their patients treated for burnout (n=632)

|  | **Univariate model^1^** | |  | **Multivariate model^2^** | |
| --- | --- | --- | --- | --- | --- |
| **Independent variables** | **OR [95% CI]** | **p-value** |  | **OR [95% CI]** | **p-value** |
| **Age group** |  |  |  |  |  |
| Less than 40 years | 1.00 | Ref |  | 1.00 | Ref |
| Between 40 and 59 years | 0.99 [0.58 - 1.67] | 0.965 |  | 1.12 [0.4 - 1.99] | 0.687 |
| 60 years and older | 0.73 [0.42 - 1.26] | 0.258 |  | 0.89 [0.48 - 1.65] | 0.716 |
| **Sex** |  |  |  |  |  |
| Male | 1.00 | Ref |  | 1.00 | Ref |
| Female | 1.26 [0.91 - 1.73] | 0.162 |  | 1.14 [0.80 - 1.62] | 0.483 |
| **Specialty** |  |  |  |  |  |
| General physician | 1.00 | Ref |  | 1.00 | Ref |
| Psychiatrist | 0.55 [0.39 - 0.76] | <0.001 |  | 0.64 [0.41- 1.00] | 0.051 |
| Other physician | 0.37 [0.19 - 0.74] | 0.005 |  | 0.35 [0.16 - 0.73] | 0.005 |
| **Principal Swiss region** |  |  |  |  |  |
| Lake Geneva region (VD, VS, GE) | 1.00 | Ref |  | 1.00 | Ref |
| Espace Mittelland (BE, FR, SO, NE, JU) | 0.86 [0.54 - 1.36] | 0.520 |  | 0.76 [0.47 - 1.25] | 0.283 |
| Northwestern Switzerland (BS, BL, AG) | 0.59 [0.35 - 0.98] | 0.041 |  | 0.69 [0.39 - 1.23] | 0.206 |
| Zürich (ZH) | 0.51 [0.29 - 0.88] | 0.016 |  | 0.52 [0.29 - 0.93] | 0.029 |
| Eastern Switzerland (GL, SH, AR, AI, SG, GR, TG) | 0.47 [0.27 - 0.81] | 0.007 |  | 0.47 [0.26 - 0.85] | 0.012 |
| Central Switzerland (LU, UR, SZ, OW, NW, ZG) | 1.18 [0.53 - 2.64] | 0.686 |  | 1.36 [0.59 - 3.15] | 0.468 |
| Ticino (TI) | 0.30 [0.11 - 0.81] | 0.017 |  | 0.29 [0.10 - 0.80] | 0.018 |
| **No of consultations** | 1.00 [1.00 - 1.00] | 0.078 |  | 1.00 [1.00 - 1.00] | 0.721 |
| **Waiting time for consultation** |  |  |  |  |  |
| Relatively fast, less than one month | 1.00 | Ref |  | 1.00 | Ref |
| More than one month | 0.52 [0.33 - 0.82] | 0.005 |  | 0.62 [0.38 - 1.01] | 0.053 |
| More than three months | 0.90 [0.42 - 1.92] | 0.788 |  | 1.05 [0.47 - 2.36] | 0.907 |
| **Treatment option** |  |  |  |  |  |
| Prescription of treatment | 1.00 | Ref |  | 1.00 | Ref |
| Contact in addition to prescription | 0.62 [0.42 - 0.93] | 0.019 |  | 0.84 [0.54 - 1.31] | 0.441 |
| Collaboration in addition to prescription | 0.96 [0.63 - 1.48] | 0.859 |  | 0.75 [0.47 - 1.19] | 0.218 |
| Contact and collaboration in addition to prescription | 0.81 [0.43 - 1.52] | 0.512 |  | 0.81 [0.41 - 1.57] | 0.528 |

1-Logistic regression model with proportion of patients that return to work (Cat: <75%/>75%), Reference: >75%) as dependent variable; 2-Logistic regression model with proportion of patients that return to work as dependent variable, adjusted for all co-variables examined in the univariate analysis
