# Supplementary material for: Diagnosis and treatment of occupational burnout in the Swiss outpatient sector: A national survey of healthcare professionals’ attributes and attitudes
Source: PLoS One. 2024 Dec 11;19(12):e0294834. doi: 10.1371/journal.pone.0294834 (PMC11633953; doi:10.1371/journal.pone.0294834)
Supplement: S12 Table — (DOCX) [file pone.0294834.s012.docx]

S12 Table. Psychologists' personnel and professional attributes associated with the highest reported proportion of return to work among their patients treated for burnout (n=306)

|  | **Univariate model^1^** | |  | **Multivariate model^2^** | |
| --- | --- | --- | --- | --- | --- |
| **Independent variables** | **OR [95% CI]** | **p-value** |  | **OR [95% CI]** | **p-value** |
| **Age group** |  |  |  |  |  |
| Less than 40 years | 1.00 | Ref |  | 1.00 | Ref |
| Between 40 and 59 years | 1.80 [1.05 - 3.08] | 0.032 |  | 2.00 [1.10 - 3.64] | 0.024 |
| 60 years and older | 1.35 [0.71 - 2.57] | 0.359 |  | 1.23 [0.58 - 2.61] | 0.587 |
| **Sex** |  |  |  |  |  |
| Male | 1.00 | Ref |  | 1.00 | Ref |
| Female | 0.94 [0.53 - 1.67] | 0.835 |  | 0.90 [0.47 - 1.72] | 0.744 |
| **Specialization** |  |  |  |  |  |
| Clinical psychologist | 1.00 | Ref |  | 1.00 | Ref |
| Psychologist-Psychotherapist | 2.51 [1.18 - 5.35] | 0.017 |  | 2.87 [1.25 - 6.59] | 0.013 |
| Other psychologist | 3.92 [1.21 - 12.67] | 0.022 |  | 3.22 [0.88 - 11.81] | 0.077 |
| **Principal Swiss region** |  |  |  |  |  |
| Lake Geneva region (VD, VS, GE) | 1.00 | Ref |  | 1.00 | Ref |
| Espace Mittelland (BE, FR, SO, NE, JU) | 0.84 [0.44 - 1.60] | 0.589 |  | 0.84 [0.41 - 1.75] | 0.641 |
| Northwestern Switzerland (BS, BL, AG) | 0.49 [0.23 - 1.02] | 0.057 |  | 0.48 [0.20 - 1.14] | 0.097 |
| Zürich (ZH) | 0.72 [0.36 - 1.41] | 0.343 |  | 0.81 [0.37 - 1.76] | 0.591 |
| Eastern Switzerland (GL, SH, AR, AI, SG, GR, TG) | 0.78 [0.29 - 2.06] | 0.609 |  | 0.97 [0.33 - 2.88] | 0.955 |
| Central Switzerland (LU, UR, SZ, OW, NW, ZG) | 0.72 [0.30 - 1.74] | 0.471 |  | 0.81 [0.30 - 2.18] | 0.673 |
| Ticino (TI) | 0.55 [0.14 - 2.22] | 0.404 |  | 0.50 [0.11 - 2.23] | 0.367 |
| **No of consultations** | 1.00 [0.99 - 1.00] | 0.347 |  | 1.00 [0.99 - 1.00] | 0.335 |
| **Financial accessibility to treatment** |  |  |  |  |  |
| Accessible for all incomes | 1.00 | Ref |  | 1.00 | Ref |
| Can be inaccessible for people with low income | 1.51 [0.96 - 2.39] | 0.077 |  | 1.39 [0.84 - 2.31] | 0.197 |
| **Waiting time for consultation** |  |  |  |  |  |
| Relatively fast, less than one month | 1.00 | Ref |  | 1.00 | Ref |
| More than one month | 0.71 [0.43 - 1.18] | 0.184 |  | 0.74 [0.42 - 1.29] | 0.286 |
| More than three months | 0.96 [0.41 - 2.27] | 0.924 |  | 1.05 [0.40 - 2.75] | 0.916 |
| **Treatment option** |  |  |  |  |  |
| Psychotherapy | 1.00 | Ref |  | 1.00 | Ref |
| Contact in addition to therapy | 0.69 [0.40 - 1.20] | 0.196 |  | 0.77 [0.41 - 1.44] | 0.416 |
| Collaboration in addition to therapy | 0.45 [0.21 - 0.94] | 0.034 |  | 0.50 [0.22 - 1.13] | 0.095 |
| Contact and collaboration in addition to therapy | 0.73 [0.34 - 1.56] | 0.414 |  | 0.96 [0.41 - 2.26] | 0.926 |

1-Logistic regression model with proportion of patients that return to work (Cat: <75%/>75%), Reference: >75%) as dependent variable; 2-Logistic regression model with proportion of patients that return to work as dependent variable, adjusted for all co-variables examined in the univariate analysis
