# Supplementary material for: Diagnosis and treatment of occupational burnout in the Swiss outpatient sector: A national survey of healthcare professionals’ attributes and attitudes
Source: PLoS One. 2024 Dec 11;19(12):e0294834. doi: 10.1371/journal.pone.0294834 (PMC11633953; doi:10.1371/journal.pone.0294834)
Supplement: S13 Table — (DOCX) [file pone.0294834.s013.docx]

S13 Table. Physicians' personnel and professional attributes associated with high (more than 25%) reported proportion of relapsed patients (n=504)

1-Logistic regression model with proportion of relapse (<25% versus >25%(Reference) as dependent variable, 2-Logistic regression model with proportion of relapse as dependent variable, adjusted for all co-variables examined in the univariate analysis
