# Supplementary material for: Diagnosis and treatment of occupational burnout in the Swiss outpatient sector: A national survey of healthcare professionals’ attributes and attitudes
Source: PLoS One. 2024 Dec 11;19(12):e0294834. doi: 10.1371/journal.pone.0294834 (PMC11633953; doi:10.1371/journal.pone.0294834)
Supplement: S15 Table — (DOCX) [file pone.0294834.s015.docx]

S15 Table. Physicians' characteristics and beliefs on the prognosis of burnout associated with reported burnout treatment modalities (n=665)

1-Multinomial logistic regression model with treatment options for burnout (Reference: only prescribing a treatment) as dependent variable; 2-Multinomial logistic regression model with treatment options for burnout, adjusted for all co-variables examined in the univariate analysis
