# Supplementary material for: Diagnosis and treatment of occupational burnout in the Swiss outpatient sector: A national survey of healthcare professionals’ attributes and attitudes
Source: PLoS One. 2024 Dec 11;19(12):e0294834. doi: 10.1371/journal.pone.0294834 (PMC11633953; doi:10.1371/journal.pone.0294834)
Supplement: S17 Table — (DOCX) [file pone.0294834.s017.docx]

S17 Table. Associations of caregivers’ specialty / specialization and choice of the treatment options with reporting of severe burnout among their patients / clients

1-Logistic regression model with severity of burnout (Cat: mild-moderate/severe, Reference: severe burnout) as dependent variable; 2-Logistic regression model with severity of burnout as dependent variable, adjusted for all co-variables examined in the univariate analysis; *the categories "General and Occupational physician" and "Psychiatrist and Occupational Physician" were omitted because of small observation number; **the category "Social psychologist" was omitted because of small number of observations
