# Supplementary material for: Diagnosis and treatment of occupational burnout in the Swiss outpatient sector: A national survey of healthcare professionals’ attributes and attitudes
Source: PLoS One. 2024 Dec 11;19(12):e0294834. doi: 10.1371/journal.pone.0294834 (PMC11633953; doi:10.1371/journal.pone.0294834)
Supplement: S18 Table — (DOCX) [file pone.0294834.s018.docx]

 S18 Table. Association of interprofessional contacts and collaborations with reported proportions of return to work and relapses among patients/clients treated for burnout

1-Logistic regression model with proportion of clients that return to work (Cat: <75%/>75%), Reference: >75%) as dependent variable; 2-Logistic regression model with proportion of clients that return to work as dependent variable, adjusted for all co-variables examined in the univariate analysis and for age, sex, specialization and region; 3-Logistic regression model with proportion of clients that relapse (Cat: <25%/>25%), Reference: >25%) as dependent variable; 4-Logistic regression model with proportion of clients that relapse as dependent variable, adjusted for all co-variables examined in the univariate analysis and for age, sex, specialization and region


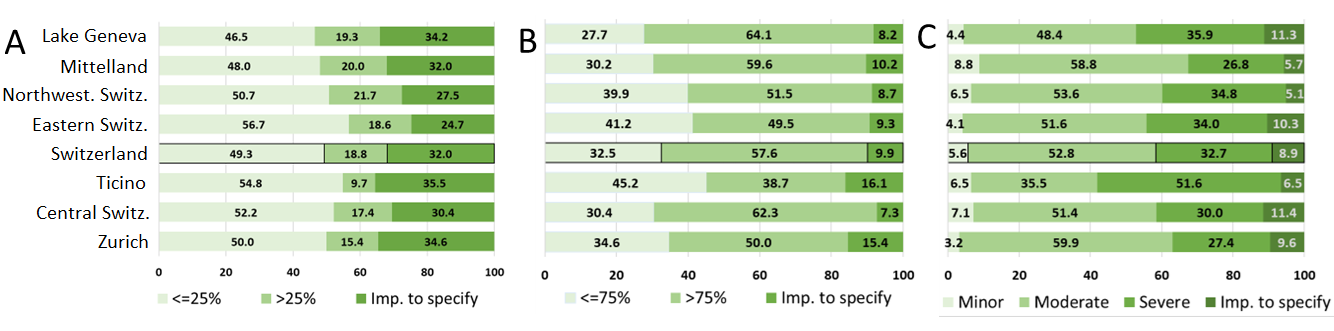


S1 Figure. Regional variation in the distribution of studies outcomes reported by health professionals who treat burned-out patients / clients (%)

A – proportion of treated patients / clients who relapse; B – proportion of patients / clients who can return to work; C – burnout severity in the majority of patients / clients


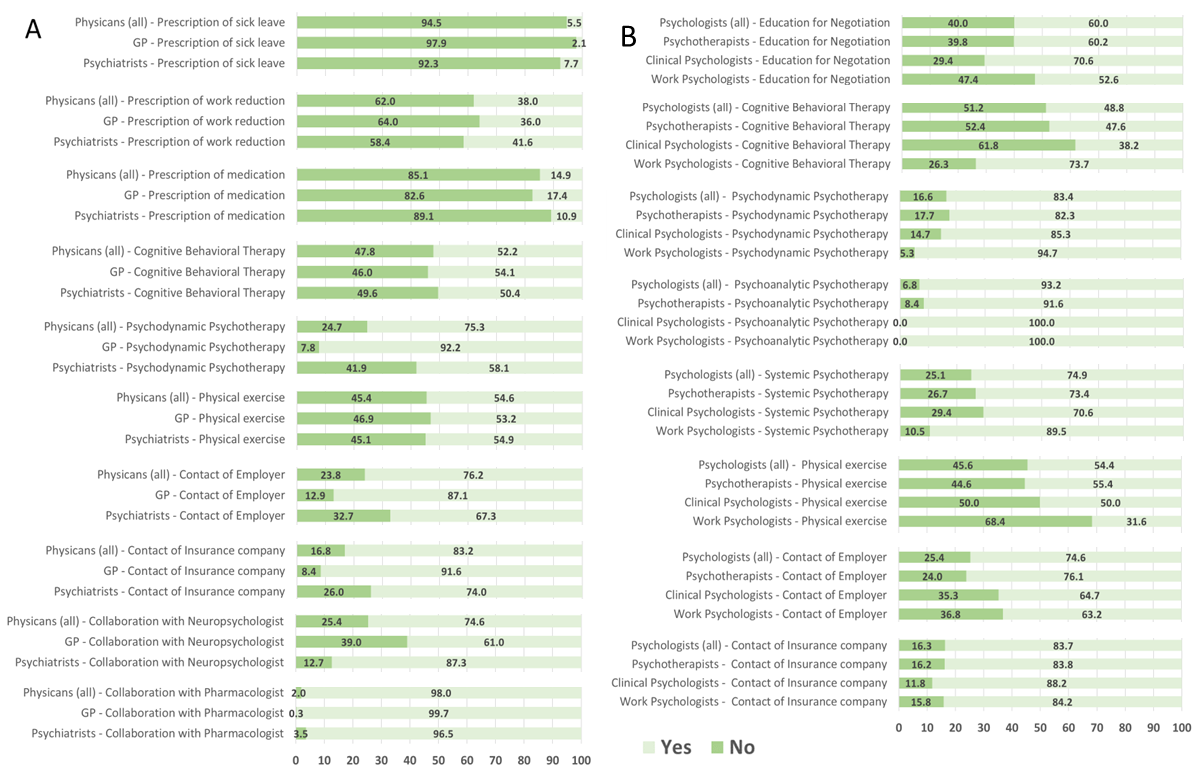


S2 Figure. Distribution (in %) of burnout treatment options by Swiss health professionals

A – Physicians and medical specialties; B – Psychologists and specializations
