# Supplementary material for: Diagnosis and treatment of occupational burnout in the Swiss outpatient sector: A national survey of healthcare professionals’ attributes and attitudes
Source: PLoS One. 2024 Dec 11;19(12):e0294834. doi: 10.1371/journal.pone.0294834 (PMC11633953; doi:10.1371/journal.pone.0294834)
Supplement: S1 Fig — (DOCX) [file pone.0294834.s019.docx]

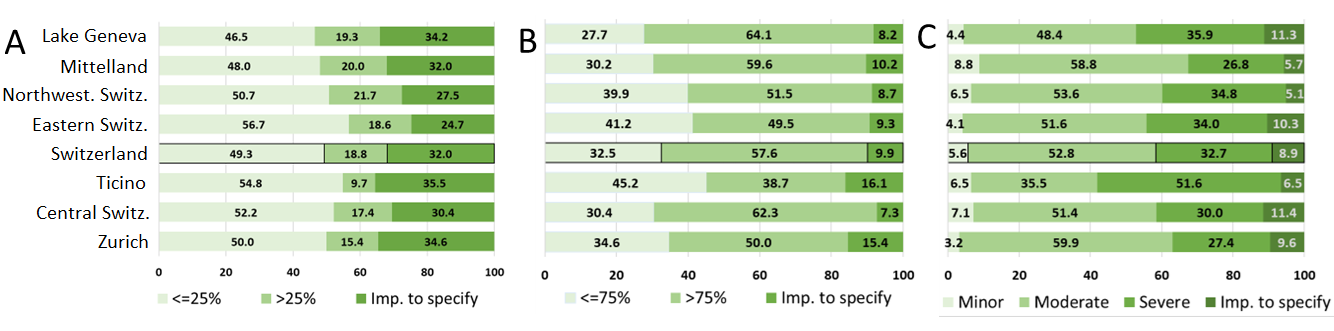


S1 Figure. Regional variation in the distribution of studies outcomes reported by health professionals who treat burned-out patients / clients (%)

A – proportion of treated patients / clients who relapse; B – proportion of patients / clients who can return to work; C – burnout severity in the majority of patients / clients
