# Supplementary material for: Diagnosis and treatment of occupational burnout in the Swiss outpatient sector: A national survey of healthcare professionals’ attributes and attitudes
Source: PLoS One. 2024 Dec 11;19(12):e0294834. doi: 10.1371/journal.pone.0294834 (PMC11633953; doi:10.1371/journal.pone.0294834)
Supplement: S2 Fig — (DOCX) [file pone.0294834.s020.docx]

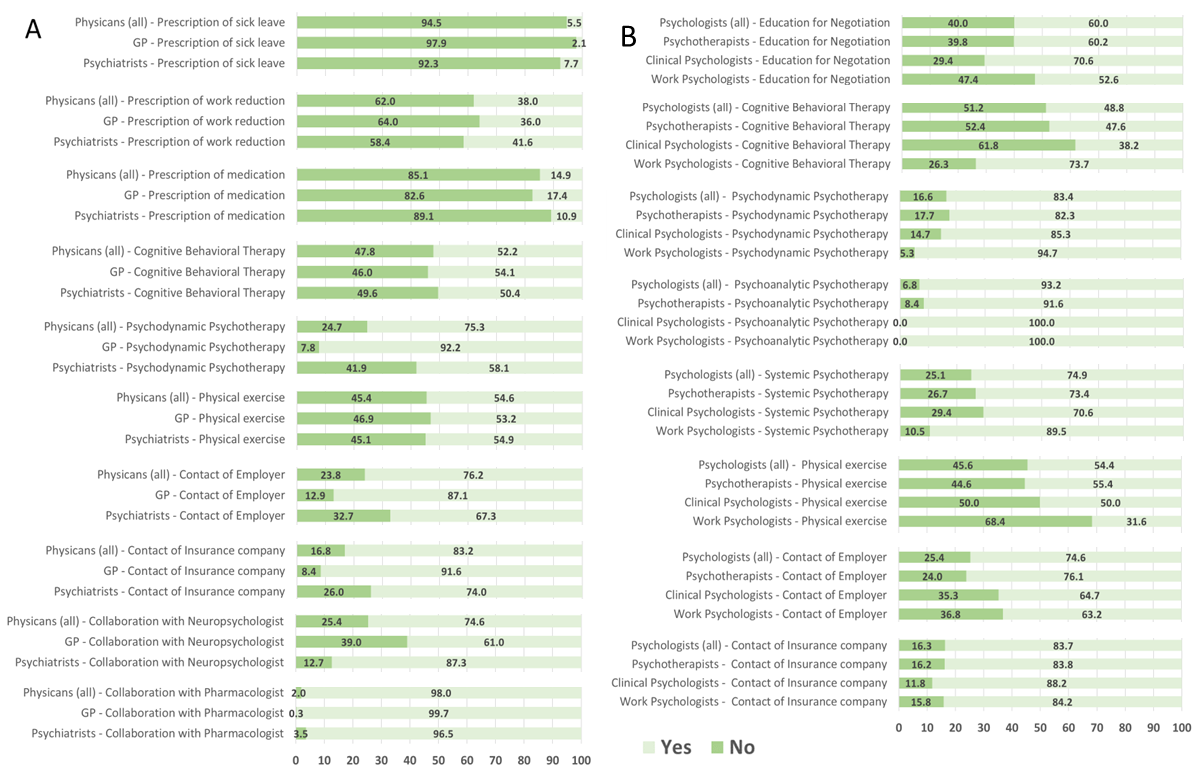


S2 Figure. Distribution (in %) of burnout treatment options by Swiss health professionals

A – Physicians and medical specialties; B – Psychologists and specializations
